# Reduced Risk for Mpox After Receipt of 1 or 2 Doses of JYNNEOS Vaccine Compared with Risk Among Unvaccinated Persons — 43 U.S. Jurisdictions, July 31–October 1, 2022

**DOI:** 10.15585/mmwr.mm7149a5

**Published:** 2022-12-09

**Authors:** Amanda B. Payne, Logan C. Ray, Matthew M. Cole, Michelle Canning, Kennedy Houck, Hazel J. Shah, Jennifer L. Farrar, Nathaniel M. Lewis, Amy Fothergill, Elizabeth B. White, Leora R. Feldstein, Lauren E. Roper, Florence Lee, Jennifer L. Kriss, Emily Sims, Ian H. Spicknall, Yoshinori Nakazawa, Adi V. Gundlapalli, Tom Shimabukuro, Adam L. Cohen, Margaret A. Honein, Jonathan Mermin, Daniel C. Payne

**Affiliations:** ^1^CDC Mpox Emergency Response Team; ^2^Epidemic Intelligence Service, CDC.

As of October 28, 2022, a total of 28,244* monkeypox (mpox) cases have been reported in the United States during an outbreak that has disproportionately affected gay, bisexual, and other men who have sex with men (MSM) (*1*). JYNNEOS vaccine (Modified Vaccinia Ankara vaccine, Bavarian Nordic), administered subcutaneously as a 2-dose (0.5 mL per dose) series (with doses administered 4 weeks apart), was approved by the Food and Drug Administration (FDA) in 2019 to prevent smallpox and mpox disease (*2*); an FDA Emergency Use Authorization issued on August 9, 2022, authorized intradermal administration of 0.1 mL per dose, increasing the number of persons who could be vaccinated with the available vaccine supply^†^ (*3*). A previous comparison of mpox incidence during July 31–September 3, 2022, among unvaccinated, but vaccine-eligible men aged 18–49 years and those who had received ≥1 JYNNEOS vaccine dose in 32 U.S. jurisdictions, found that incidence among unvaccinated persons was 14 times that among vaccinated persons (95% CI = 5.0–41.0) (*4*). During September 4–October 1, 2022, a total of 205,504 persons received JYNNEOS vaccine dose 2 in the United States.^§^ To further examine mpox incidence among persons who were unvaccinated and those who had received either 1 or 2 JYNNEOS doses, investigators analyzed data on 9,544 reported mpox cases among men^¶^ aged 18–49 years during July 31–October 1, 2022, from 43 U.S. jurisdictions,** by vaccination status. During this study period, mpox incidence (cases per 100,000 population at risk) among unvaccinated persons was 7.4 (95% CI = 6.0–9.1) times that among persons who received only 1 dose of JYNNEOS vaccine ≥14 days earlier and 9.6 (95% CI = 6.9–13.2) times that among persons who received dose 2 ≥14 days earlier. The observed distribution of subcutaneous and intradermal routes of administration of dose 1 among vaccinated persons with mpox was not different from the expected distribution. This report provides additional data suggesting JYNNEOS vaccine provides protection against mpox, irrespective of whether the vaccine is administered intradermally or subcutaneously. The degree and durability of such protection remains unclear. Persons eligible for mpox vaccination should receive the complete 2-dose series to optimize strength of protection^††^ (*5*).

Aggregate weekly numbers of confirmed and probable mpox cases^§§^ among men aged 18–49 years with illness onset (i.e., earliest date available^¶¶^) during July 31–October 1, 2022, were analyzed across 43 public health jurisdictions.*** These jurisdictions routinely ascertain patient vaccination status (receipt of ≥1 dose of JYNNEOS vaccine) and route of vaccine administration through interviews and immunization registries and submit deidentified vaccine administration data to CDC. Persons with mpox were categorized as 1) unvaccinated^†††^; 2) vaccinated, with illness onset ≥14 days after administration of dose 1 and before or <14 days after receipt of dose 2; or 3) vaccinated with illness onset ≥14 days after dose 2. Persons with illness onset <14 days after receipt of dose 1, potentially vaccinated persons (possibly vaccinated but without dose number or documented date of vaccination), and persons vaccinated before 2022 were excluded.

One- and 2-dose vaccination coverage was estimated as the total number of persons vaccinated as of 2 weeks before the end date of a week divided by the estimated vaccine-eligible population aged 18–49 years, including persons in each jurisdiction who might benefit from vaccination in the context of the outbreak (estimated as the number of men who are either MSM living with HIV acquired through male-to-male sexual contact, injection drug use, or both, or who are eligible for HIV preexposure prophylaxis [HIV-PrEP])^§§§^ (*6*). The number of eligible unvaccinated persons was obtained by subtracting the number of vaccinated persons from jurisdiction-specific estimates of the vaccine-eligible population. Weekly incidence^¶¶¶^ by vaccination status was estimated as the number of cases divided by the number of persons either eligible but unvaccinated as of that week or vaccinated as of 2 weeks earlier.**** Weekly incidence among persons receiving dose 2 was estimated for September 4–October 1, 2022, when population coverage with 2 vaccine doses among the total eligible population was nearly 5%. The incidence rate ratio (IRR) during the study period was calculated using negative binomial regression, controlling for week in the model using an indicator variable, which is a modified approach to that used in previous analyses (*4*).

A supplementary analysis was conducted estimating the effect of dose 1 and dose 2. A Cox proportional hazards regression analysis that accounted for follow-up time among unvaccinated persons compared with that among persons known to have received either 1 or both vaccine doses was used.

The observed distribution of subcutaneous and intradermal routes of administration of dose 1 among vaccinated persons with mpox was compared with the expected distribution^††††^ among all vaccinated persons, based on vaccine administration records in 14 jurisdictions with complete route of administration data for ≥80% of reported vaccinated persons with mpox.

SAS (version 9.4; SAS Institute) and R (version 4.0.3; R Foundation) were used to conduct all analyses. This activity was reviewed by CDC and was conducted consistent with applicable federal law and CDC policy.^§§§§^

During July 31–October 1, 2022, in 43 jurisdictions reporting 11,581 mpox cases (range across jurisdictions = 2–3,424 cases), a total of 9,544 (82.4%) were reported among men aged 18–49 years (Table 1). Among these cases, 8,320 (87.2%) occurred in unvaccinated persons and 1,224 (12.8%) in vaccinated persons, including 218 (17.8%) in persons without a known vaccination date. Among cases in vaccinated persons whose vaccination date was known, 614 (61%) were in persons whose illness onset occurred ≤13 days after receipt of dose 1 and 392 (39%) in persons with illness onset ≥14 days after receipt of dose 1; among this group, 48 cases (12.2%) (0.5% of all cases) were among persons with illness onset ≥14 days after receipt of dose 2. Population coverage with ≥1 vaccine dose received ≥14 days before the end of each week increased from 5.7% (July 31) to 45.5% (September 25); 2-dose coverage increased from 0.1% to 17%.

**TABLE 1:** Mpox cases among men* aged 18–49 years, by vaccination status,^†^ and JYNNEOS vaccination coverage, by week (N = 9,544) — 43 U.S. jurisdictions,^§^^,^^¶^ July 31–October 1, 2022

Characteristic	No. (%) by week beginning									Total
	Jul 31	Aug 7	Aug 14	Aug 21	Aug 28	Sep 4	Sep 11	Sep 18	Sep 25	
Total mpox cases**	**1,823**	**1,649**	**1,450**	**1,250**	**1,035**	**854**	**605**	**494**	**384**	**9,544**
**Vaccination status**										
Unvaccinated	1,621 (88.9)	1,422 (86.2)	1,250 (86.2)	1,068 (85.4)	889 (85.9)	744 (87.1)	546 (90.2)	440 (89.1)	340 (88.5)	**8,320 (87.2)**
Vaccinated	202 (11.1)	227 (13.8)	200 (13.8)	182 (14.6)	146 (14.1)	110 (12.9)	59 (9.8)	54 (10.9)	44 (11.5)	**1,224 (12.8)**
**Vaccination date known (n = 1,224)**										
No	40 (19.8)	30 (13.2)	31 (15.5)	36 (19.8)	25 (17.1)	24 (21.8)	9 (15.3)	10 (18.5)	13 (29.5)	**218 (17.8)**
Yes	162 (80.2)	197 (86.8)	169 (84.5)	146 (80.2)	121 (82.9)	86 (78.2)	50 (84.7)	44 (81.5)	31 (70.5)	**1,006 (82.2)**
**Illness onset relative to dose 1 of vaccination^††^ (n = 1,006)**										
0–13 days after dose 1	141 (87)	145 (73.6)	112 (66.3)	86 (58.9)	62 (51.2)	30 (34.9)	24 (48)	9 (20.5)	5 (16.1)	**614 (61)**
≥14 days after dose 1	21 (13)	52 (26.4)	57 (33.7)	60 (41.1)	59 (48.8)	56 (65.1)	26 (52)	35 (79.5)	26 (83.9)	**392 (39)**
**Illness onset relative to dose 2 of vaccination^††^ (n = 392)**										
Before dose 2	21 (100)	48 (92.3)	50 (87.7)	46 (76.7)	47 (79.7)	36 (64.3)	13 (50)	18 (51.4)	16 (61.5)	**295 (75.3)**
0–13 days after dose 2	0 (—)	4 (7.7)	4 (7.0)	11 (18.3)	8 (13.6)	8 (14.3)	7 (26.9)	6 (17.1)	1 (3.8)	**49 (12.5)**
≥14 days after dose 2	0 (—)	0 (—)	3 (5.3)	3 (5.0)	4 (6.8)	12 (21.4)	6 (23.1)	11 (31.4)	9 (34.6)	**48 (12.2)**
**JYNNEOS vaccination coverage (%)**										
1 dose^§§^	5.7	10.4	16.9	24.6	30.9	36.2	40.2	42.9	45.5	**NA**
2 dose^¶¶^	0.1	0.2	0.3	0.8	2.1	4.7	8.4	12.7	17	**NA**

**Abbreviations**: mpox = monkeypox; NA = not applicable; PCR = polymerase chain reaction.

* Sex assigned at birth or gender identity.

^†^ Vaccinated is defined as receipt of ≥1 dose of JYNNEOS vaccine (1-dose = receipt of dose 1 ≥14 days earlier; 2-dose = receipt of dose 2 ≥14 days earlier).

^§^ Alabama, Alaska, California, Colorado, Connecticut, District of Columbia, Florida, Georgia, Hawaii, Idaho, Illinois, Iowa, Kansas, Kentucky, Louisiana, Maine, Maryland, Massachusetts, Michigan, Minnesota, Mississippi, Missouri, Montana, Nevada, New Hampshire, New Mexico, New York (excluding New York City), North Dakota, Ohio, Oklahoma, Oregon, Pennsylvania, Puerto Rico, Rhode Island, South Carolina, South Dakota, Tennessee, Utah, Vermont, Virginia, West Virginia, Wisconsin, and Wyoming.

^¶^ Jurisdictions were included if age and sex assigned at birth or gender identity was available for ≥70% of cases reported, vaccination status was available for ≥50% of cases, or jurisdiction confirmed cases were linked to immunization registry entries, and deidentified vaccination administration data were submitted to CDC.

** Confirmed (presence of *Monkeypox virus* DNA by PCR testing or next-generation sequencing of a clinical specimen or isolation of *Monkeypox virus* in culture from a clinical specimen) and probable (presence of *Orthopoxvirus* DNA by PCR testing, or *Orthopoxvirus* using immunohistochemical or electron microscopy or detectable levels of anti-*Orthopoxvirus* immunoglobulin M antibody) mpox cases.

^††^ Among those with known vaccination date.

^§§^ Proportion of population eligible for vaccination who had received 1 dose of JYNNEOS vaccine as of 2 weeks before the end of the week. This underlying population included persons in each jurisdiction who might benefit from expanded vaccination in the context of the outbreak and was estimated as the number of gay, bisexual, and other men who have sex with men who have HIV infection or who are eligible to receive HIV preexposure prophylaxis.

^††^ Proportion of population eligible for vaccination who had received 2 doses of JYNNEOS vaccine as of 2 weeks before the end of the week. This underlying population included persons in each jurisdiction who might benefit from expanded vaccination in the context of the outbreak and was estimated as the number of gay, bisexual, and other men who have sex with men who have HIV infection or who are eligible to receive HIV preexposure prophylaxis.

Mpox incidence estimates were higher among unvaccinated persons than among persons known to have received only 1 dose of JYNNEOS vaccine ≥14 days earlier (IRR = 7.4; 95% CI = 6.0–9.1) and among those who received dose 2 ≥14 days earlier (IRR = 9.6; 95% CI = 6.9–13.2) (Figure). A supplementary analysis using a Cox proportional hazards model to account for follow-up time also indicated that incidence was higher among unvaccinated persons than among persons known to have only received dose 1 (hazard ratio = 4.3; 95% CI = 3.9–4.8) and those who received 2 doses (hazard ratio = 7.6; 95% CI = 5.7–10.2), although the strength of the effect was somewhat attenuated compared with the primary analysis (Supplementary Table, https://stacks.cdc.gov/view/cdc/122452).

**FIGURE:**
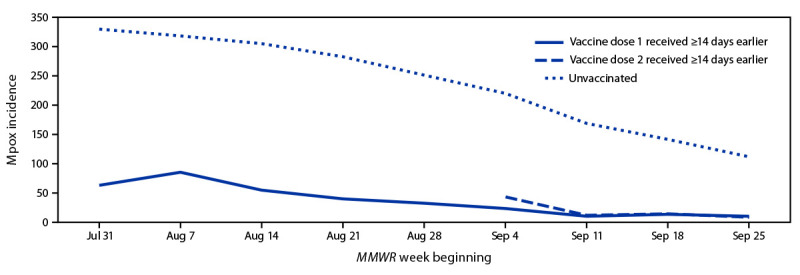
Weekly mpox incidence* among vaccine-eligible^†^ men aged 18–49 years, by vaccination status^§^ — 43 U.S. jurisdictions,^¶^^,^** July 31–October 1, 2022 **Abbreviation:** IRR = incidence rate ratio. * Cases per 100,000 population. Rate in vaccinated persons = number of probable or confirmed cases reported to CDC with date of illness onset, specimen collection, lab test completion, admission, diagnosis, discharge, case investigation start date, or date first electronically submitted or reported to the county, state, or public health department (earliest available date) ≥14 days after receiving dose 1 or dose 2 of JYNNEOS vaccine among total vaccinated population as of 2 weeks previously. Rate in unvaccinated persons = number of probable or confirmed cases reported to CDC without evidence of vaccination among total unvaccinated population. ^†^ Gay, bisexual, and other men who have sex with men who have HIV infection or who are eligible to receive HIV preexposure prophylaxis were considered eligible for vaccination. ^§^ IRR comparing unvaccinated persons with those who received only 1 dose of vaccine ≥14 days earlier was 7.4. IRR comparing unvaccinated persons with those who received dose 2 of vaccine ≥14 days earlier was 9.6. ^¶^ Alabama, Alaska, California, Colorado, Connecticut, District of Columbia, Florida, Georgia, Hawaii, Idaho, Illinois, Iowa, Kansas, Kentucky, Louisiana, Maine, Maryland, Massachusetts, Michigan, Minnesota, Mississippi, Missouri, Montana, Nevada, New Hampshire, New Mexico, New York (excluding New York City), North Dakota, Ohio, Oklahoma, Oregon, Pennsylvania, Puerto Rico, Rhode Island, South Carolina, South Dakota, Tennessee, Utah, Vermont, Virginia, West Virginia, Wisconsin, and Wyoming. ** Jurisdictions were included if age and sex assigned at birth or gender identity was available for ≥70% of cases reported, vaccination status was available for ≥50% of cases, or jurisdiction-confirmed cases were linked to immunization registry entries, and deidentified vaccination administration data were submitted to CDC.

Among persons with illness onset ≥14 days after vaccine dose 1, 263 (87.1%) and 39 (12.9%) had received subcutaneous and intradermal administration, respectively. The proportion of vaccinated persons with mpox known to have received dose 1 subcutaneously or intradermally was not statistically different from that of the overall vaccinated population (83% and 17%, respectively) (p = 0.28) (Table 2).

**TABLE 2:** Route of JYNNEOS vaccine administration among persons with mpox* vaccinated^†^ ≥14 days before illness onset^§^ compared with expected proportions^¶^ — 14 U.S. jurisdictions,**^,^^††^ July 31–October 1, 2022

Route of vaccine administration^§§^	Observed no. (%)	Expected no. (%)	p-value^¶¶^
Subcutaneous	263 (87.1)	253 (83.0)	0.28
Intradermal	39 (12.9)	52 (17.0)	

**Abbreviations**: mpox = monkeypox; PCR = polymerase chain reaction.

* Confirmed (presence of *Monkeypox virus* DNA by PCR testing or next-generation sequencing of a clinical specimen or isolation of *Monkeypox virus* in culture from a clinical specimen) and probable (presence of *Orthopoxvirus* DNA by PCR testing, or *Orthopoxvirus* using immunohistochemical or electron microscopy or detectable levels of anti-*Orthopoxvirus* immunoglobulin M antibody) mpox cases.

^†^ Receipt of ≥1 dose of JYNNEOS vaccine.

^§^ Earliest date available for each case; might include illness onset, specimen collection, laboratory test completion, admission, diagnosis, discharge, case investigation start date, or date first electronically submitted or reported to the county, state, or public health department.

^¶^ Based on vaccine administration data submitted by participating jurisdictions. The distribution of route of administration among vaccinated persons was summarized by week and applied to weekly case counts.

** California, Connecticut, District of Columbia, Florida, Illinois, Kansas, Louisiana, Massachusetts, Minnesota, New Mexico, New York (excluding New York City), Tennessee, Utah, and Wisconsin.

^††^ Jurisdictions were included if age and sex assigned at birth or gender identity was available for ≥70% of cases reported, vaccination status was available for ≥50% of cases in men (defined by either sex assigned at birth or gender identity) aged 18–49 years or the jurisdiction confirmed cases are linked to immunization registry entries, route of administration was complete for >80% of cases occurring ≥14 days after receipt of dose 1, and deidentified vaccine administration data were submitted to CDC.

^§§^ Limited to persons vaccinated ≥14 days before illness onset with recorded route of administration as subcutaneous or intradermal. One person with mpox who was vaccinated ≥14 days before illness onset was reported to have another route of vaccine administration. Route of administration was unknown for 39 persons with mpox who were vaccinated ≥14 days before illness onset; 33 persons who received dose 1 on or before August 9, 2022, were assumed to have received subcutaneous vaccination.

^¶¶^ Pearson’s chi-square test comparing observed and expected proportions of route of administration among cases with approved or authorized route of administration.

## Discussion

In this evaluation of mpox among men aged 18–49 years, incidences were lower among those who were vaccinated than among unvaccinated, vaccine-eligible persons. A proportional hazards model that accounted for time-varying risk supported the finding of a larger risk reduction among persons who had received 2 vaccine doses than among those who had received only 1 dose. Compared with a previous report (*4*), this analysis expands knowledge about mpox incidence by vaccination status by including more jurisdictions during a longer observation period, resulting in the addition of the equivalent of >1 million person-weeks of follow-up. Further, increased completeness of vaccination administration date (from 43% to 82% completeness) and a better-fitting statistical model yielded more precise effect estimates. These findings are consistent with those of the previous analysis as well as recent studies reporting some protection (*7*) and modest induction of antibody levels (*8*,*9*) after the first JYNNEOS vaccine dose.

The analysis also suggested no difference in vaccine performance between subcutaneous and intradermal administration. This supports previous clinical trial data that indicated similar immune responses to JYNNEOS vaccination over time after intradermal or subcutaneous administration (*10*).

The findings in this report are subject to at least five limitations. First, linkage of mpox case surveillance and vaccination administration data might have resulted in misclassifications that could influence estimates. This approach assumed that persons with unknown vaccination status were unvaccinated and excluded those with unknown date of vaccination, because timing between vaccination and illness onset could not be established, potentially underestimating incidence among vaccinated persons. Second, this analysis was unable to control for possible differences in testing or behaviors that affect the risk for *Monkeypox virus* exposure (e.g., reducing number of sexual partners), or possible differences in risk of infection because of patient characteristics (e.g., age, underlying medical conditions, and HIV-associated immune suppression); consequently, causal attribution of these results to vaccination cannot be definitively inferred from these data. Third, temporality of exposures that result in infection is not known, nor was it possible to determine whether vaccination was administered as postexposure or preexposure prophylaxis. Fourth, confirmation that all identified persons with mpox were members of the population eligible for vaccination was not possible. Finally, considering persons vaccinated as of 2 weeks before the end date of a surveillance week could overestimate the number of persons vaccinated each week and, thus, underestimate the weekly incidence among vaccinated persons.

Monitoring mpox incidence by vaccination status using currently available surveillance data provides an indication of the real-world impact of JYNNEOS vaccine on prevention of mpox to guide rapid public health decision-making, subject to the limitations noted. Although the findings suggest a protective effect of JYNNEOS vaccination, additional epidemiologic studies that better account for potential biases will provide additional data on the magnitude and duration of protection by JYNNEOS against mpox. These findings also suggest that JYNNEOS vaccination provides protection against mpox infection, irrespective of route of administration. Persons who are eligible for mpox vaccination should receive the complete recommended 2-dose series to optimize their protection against mpox (*5*).

**Summary**

**What is already known about this topic?**

Real-world data on the magnitude and durability of protection by JYNNEOS vaccine against monkeypox (mpox) remain limited.

**What is added by this report?**

Among JYNNEOS vaccine-eligible men aged 18–49 years in 43 U.S. jurisdictions, mpox incidence among unvaccinated persons was 9.6 times as high as that among persons who had received 2 vaccine doses and 7.4 times as high as that among persons who had received only the first dose. Preliminary evidence indicates no difference in protection between subcutaneous and intradermal administration routes.

**What are the implications for public health practice?**

Although further study is needed to determine the magnitude and durability of protection, evidence indicates that JYNNEOS vaccination provides protection against mpox. Vaccine-eligible persons should complete the 2-dose vaccination series.

## Footnotes

*
https://www.cdc.gov/poxvirus/monkeypox/response/2022/us-map.html

†
https://www.hhs.gov/about/news/2022/06/28/hhs-announces-enhanced-strategy-vaccinate-protect-at-risk-individuals-from-current-monkeypox-outbreak.html

§
https://www.cdc.gov/poxvirus/monkeypox/response/2022/vaccines_data.html

¶ Persons who reported male sex assigned at birth or male gender identity.

** Alabama, Alaska, California, Colorado, Connecticut, District of Columbia, Florida, Georgia, Hawaii, Idaho, Illinois, Iowa, Kansas, Kentucky, Louisiana, Maine, Maryland, Massachusetts, Michigan, Minnesota, Mississippi, Missouri, Montana, Nevada, New Hampshire, New Mexico, New York (excluding New York City), North Dakota, Ohio, Oklahoma, Oregon, Pennsylvania, Puerto Rico, Rhode Island, South Carolina, South Dakota, Tennessee, Utah, Vermont, Virginia, West Virginia, Wisconsin, and Wyoming.

††
https://www.cdc.gov/poxvirus/monkeypox/clinicians/vaccines/vaccine-considerations.html

§§ Confirmed (presence of *Monkeypx virus* DNA by polymerase chain reaction [PCR] testing or next-generation sequencing of a clinical specimen or isolation of *Monkeypox virus* in culture from a clinical specimen) and probable (presence of *Orthopoxvirus* DNA by PCR testing, or *Orthopoxvirus* using immunohistochemical or electron microscopy or detectable levels of anti-*Orthopoxvirus* immunoglobulin M antibody) mpox cases.

¶¶ Dates available for selection varied by how the case was reported to the system: illness onset, specimen collection, laboratory test completion, admission, diagnosis, discharge, case investigation start date, or date first electronically submitted or reported to county, state, or public health department.

*** Jurisdictions were included if age and sex assigned at birth or gender identity was available for ≥70% of cases reported, vaccination status was available for ≥50% of cases, or jurisdiction-confirmed cases were linked to immunization registry entries, and deidentified vaccination administration data were submitted to CDC.

††† No evidence in case record of receipt of JYNNEOS vaccine or vaccination date after illness onset, including records for which vaccination information was unknown.

§§§ The number eligible for HIV-PrEP was estimated as the ratio of the jurisdiction-specific number of MSM receiving HIV-PrEP and the jurisdiction-specific HIV-PrEP coverage. The number of MSM with HIV or who are HIV-PrEP–eligible aged 18–49 years was estimated by aggregating 2021 U.S. Census Bureau estimates for males aged 0–12, 13–17, 18–49, and ≥50 years, calculating the state proportion for each age group, and multiplying by the estimated number of MSM with HIV or who are eligible for HIV-PrEP in each state to obtain proportional distributions. Estimates of the number of MSM with HIV infection were obtained from 2020 HIV prevalence estimates. https://www.cdc.gov/nchhstp/atlas/index.htm

¶¶¶ Cases and vaccine doses administered are aggregated by *MMWR* week (Sunday–Saturday).

**** Because most vaccine administered during the study period was postexposure prophylaxis (PEP) or expanded PEP, this time point was chosen to account for the incubation period after exposure. FDA immunogenicity data indicated antibody titers 2 weeks after dose 1 were similar to titers 4 weeks after dose 1 and were significantly higher than prevaccination antibody titers.

†††† Jurisdiction-specific distributions of cases in vaccinated persons by route of administration were obtained by summarizing the cumulative number of first vaccine doses administered to men aged 18–49 years by route of administration as of a given surveillance week. The expected number of cases in vaccinated persons by route of administration was obtained by applying the jurisdiction-specific distribution to the number of cases reported from that jurisdiction within a given *MMWR* week (e.g., expected number of cases with vaccine administered intradermally = [cumulative number of vaccines administered intradermally/total number of vaccines administered] × number of cases in vaccinated persons). The number of expected cases by route of administration was summed across jurisdictions and *MMWR* weeks.

§§§§ 5 C.F.R. part 46, 21 C.F.R. part 56; 42 U.S.C. Sect. 241(d); 5 U.S.C. Sect. 552a; 44 U.S.C. Sect. 3501 et seq.

## References

[1] Philpott D, Hughes CM, Alroy KA, ; CDC Multinational Monkeypox Response Team. Epidemiologic and clinical characteristics of monkeypox cases—United States, May 17–July 22, 2022. MMWR Morb Mortal Wkly Rep 2022;71:1018–22. 10.15585/mmwr.mm7132e3 35951487 PMC9400536

[2] Food and Drug Administration. JYNNEOS [package insert, revised June 2021]. Silver Spring, MD: US Department of Health and Human Services, Food and Drug Administration; 2021. https://www.fda.gov/media/131078/download

[3] Food and Drug Administration. Emergency use authorization for the emergency use of JYNNEOS. Silver Spring, MD: US Department of Health and Human Services, Food and Drug Administration; 2022. https://www.fda.gov/media/160774/download

[4] Payne AB, Ray LC, Kugeler KJ, Incidence of monkeypox among unvaccinated persons compared with persons receiving ≥1 JYNNEOS vaccine dose—32 U.S. jurisdictions, July 31–September 3, 2022. MMWR Morb Mortal Wkly Rep 2022;71:1278–82. 10.15585/mmwr.mm7140e3 36201401 PMC9541026

[5] Hatch GJ, Graham VA, Bewley KR, Assessment of the protective effect of Imvamune and Acam2000 vaccines against aerosolized monkeypox virus in cynomolgus macaques. J Virol 2013;87:7805–15. 10.1128/JVI.03481-12 23658452 PMC3700201

[6] Administration for Strategic Preparedness and Response. JYNNEOS vaccine distribution by jurisdiction. Washington, DC: US Department of Health and Human Services, Administration for Strategic Preparedness and Response; 2022. https://aspr.hhs.gov/SNS/Pages/JYNNEOS-Distribution.aspx

[7] Arbel R, Sagy YW, Zucker R, Effectiveness of a single-dose modified vaccinia Ankara in human monkeypox: an observational study. Research Square [Preprint posted online September 23, 2022]. https://www.researchsquare.com/article/rs-1976861/v2

[8] Food and Drug Administration. BLA clinical review memorandum. Silver Spring, MD: US Department of Health and Human Services, Food and Drug Administration; 2019. https://www.fda.gov/media/131870/download

[9] Frey SE, Wald A, Edupuganti S, Comparison of lyophilized versus liquid modified vaccinia Ankara (MVA) formulations and subcutaneous versus intradermal routes of administration in healthy vaccinia-naïve subjects. Vaccine 2015;33:5225–34. 10.1016/j.vaccine.2015.06.075 26143613 PMC9533873

[10] Frey SE, Stapleton JT, Ballas ZK, ; DMID 09-0002 MVA Vaccine Study Group. Human antibody responses following vaccinia immunization using protein microarrays and correlation with cell-mediated immunity and antibody-dependent cellular cytotoxicity responses. J Infect Dis 2021;224:1372–82. 10.1093/infdis/jiab111 33675226 PMC8861366

